# Fabrication of Flexible Microneedle Array Electrodes for Wearable Bio-Signal Recording

**DOI:** 10.3390/s18041191

**Published:** 2018-04-13

**Authors:** Lei Ren, Shujia Xu, Jie Gao, Zi Lin, Zhipeng Chen, Bin Liu, Liang Liang, Lelun Jiang

**Affiliations:** 1 Guangdong Provincial Key Laboratory of Sensor Technology and Biomedical Instrument, Sun Yat-Sen University, Guangzhou 510006, China; renlei5@mail.sysu.edu.cn (L.R.); xushj9@mail2.sysu.edu.cn (S.X.); gaoj35@mail2.sysu.edu.cn (J.G.); linz3@mail2.sysu.edu.cn (Z.L.); tiaopiji@icloud.com (Z.C.); liub78@mail.sysu.edu.cn (B.L.); 2 School of Mechanical and Automotive Engineering, South China University of Technology, Guangzhou 510640, China; melliang@scut.edu.cn

**Keywords:** laser-direct writing, magneto-rheological drawing lithography, microneedle array, electrode, bio-signal

## Abstract

Laser-direct writing (LDW) and magneto-rheological drawing lithography (MRDL) have been proposed for the fabrication of a flexible microneedle array electrode (MAE) for wearable bio-signal monitoring. Conductive patterns were directly written onto the flexible polyethylene terephthalate (PET) substrate by LDW. The microneedle array was rapidly drawn and formed from the droplets of curable magnetorheological fluid with the assistance of an external magnetic field by MRDL. A flexible MAE can maintain a stable contact interface with curved human skin due to the flexibility of the PET substrate. Compared with Ag/AgCl electrodes and flexible dry electrodes (FDE), the electrode–skin interface impedance of flexible MAE was the minimum even after a 50-cycle bending test. Flexible MAE can record electromyography (EMG), electroencephalography (EEG) and static electrocardiography (ECG) signals with good fidelity. The main features of the dynamic ECG signal recorded by flexible MAE are the most distinguishable with the least moving artifacts. Flexible MAE is an attractive candidate electrode for wearable bio-signal monitoring.

## 1. Introduction

Home health care management has attracted increasing interest in the last decades due to the rapid growth of the ageing population and the prevalence of chronic diseases. Bio-signal monitoring, including electrocardiography (ECG), electromyography (EMG), and electroencephalography (EEG), aid the understanding of human pathological and physiological conditions [1]. Therefore, it is important to develop special electrodes for wearable bio-signal monitoring in home health care. Usually, Ag/AgCl electrodes are widely used in clinical practice, which require skin preparation and the use of gel [2,3,4]. The gel may cause skin irritations and allergic reactions, and gets dry during monitoring, reducing the quality of the recorded signals [5,6]. Thus, Ag/AgCl electrodes are not suitable for long-term bio-signal monitoring in home health care. Microneedle array electrodes (MAEs) can address the above issues well. MAEs can easily pierce the skin and form a contact interface between microneedles and skin tissue, eliminating the high impedance of the stratum corneum (SC) layer and reducing the motion of the artifact [7]. However, most MAEs are fabricated on rigid substrates, making it difficult to maintain conformal contact with curved human skin. Flexible MAEs integrated with a conductive pattern are an attractive choice for wearable bio-signal monitoring.

The fabrication of conductive patterns and formation of microneedle arrays on a flexible substrate are the key process for flexible MAEs. Currently, numerous methods have been proposed to fabricate conductive patterns, including screen printing [8,9,10], inkjet printing [11,12,13,14], lithography [15,16], sputtering [17,18] and laser-direct writing (LDW) [19]. Screen printing suffers from low pattern resolution, inject printing is limited by the synthesis of conductive inks and nozzle-clogging problems [20], and lithography and sputtering has a high resolution but requires a complicated process and sophisticated equipment. LDW is a promising process for the easy fabrication of conductive patterns due to it being high resolution, maskless, low-cost and easy to manipulate. Various methods have been employed to fabricate microneedle arrays, including etching and lithography [4,21,22,23,24,25], laser machining [26,27,28], micro-molding [29,30,31], 3D printing [32], thermal drawing [33], and magnetization-induced self-assembly [34,35], but most of microneedle arrays were fabricated on the rigid substrates. These techniques cannot directly fabricate microneedle arrays on a flexible substrate. Wang et al. [36,37] fabricated a parylene-based flexible MAE for long-term bio-signal monitoring. However, the fabrication process was extremely complex. It included thermal oxidization, reactive-ion etching, the deposition of parylene films, the lift-off technique, sputtering and so on. Srivastava et al. [22] presented a polymer-photoresist-based flexible MAE for bio-signal sensing. The microneedle array was fabricated by UV maskless lithography, which required expensive and sophisticated equipment located in clean rooms. These flexible MAEs exhibited good bio-signal recording performance, but the fabrication processes were complex, resulting in a high cost. The magneto-rheological drawing lithography (MRDL) technique is a promising fabrication method that meets the above challenges. We have demonstrated that MRDL can easily draw the microneedle array from the droplets of curable magneto-rheological fluid (CMRF) on the flexible substrates under the assistance of an external magnetic field [37,38].

In this work, we present a simple fabrication process for flexible MAEs for wearable bio-signal recording. Conductive patterns are directly written on a thin polyethylene terephthalate (PET) film by LDW. The microneedle array is one-step drawn and formed on the conductive pattern by MRDL. The flexible MAE is extremely flexible and can closely conform to the skin surface. In the following work, the fabrication process of flexible MAEs will be discussed and the morphology of flexible MAEs will be characterized. The flexible MAE monitoring performance of the electrode–skin interface impedance (EII) before and after a bending test, electromyography (EMG), electroencephalography (EEG), and electrocardiography (ECG) signals are evaluated in comparison to Ag/AgCl electrodes and flexible dry electrodes (FDE).

## 2. Fabrication of Flexible MAE

**Figure 1:**
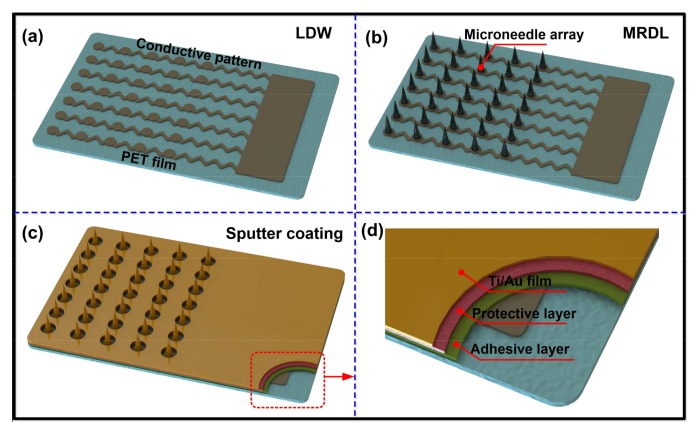
Schematic diagram of flexible MAE fabrication process. (**a**) Conductive pattern fabricated by LDW, (**b**) Microneedle array fabricated by MRDL, (**c**) Ti/Au layer coating, (**d**) The schematic of flexible MAE.

The main fabrication steps of the flexible MAEs include the preparation of silver ink, the fabrication of the conductive pattern by LDW, the fabrication of the microneedle array by MRDL, and sputter coating. The fabrication process of the flexible MAEs is shown in Figure 1.

### 2.1. Preparation of Silver Ink

Trisodium citrate dehydrate (TCD, Tianjin Zhi Yuan Chemical Reagents Factory, Tianjin, China), polyvinylpyrrolidone (PVP, Mw = 58,000, Aladdin Industrial Corporation, Shanghai, China) and silver nitrate (AgNO_3_, Mw = 169.87, Shanghai Macklin Biochemical Co., Ltd., Shanghai, China) were purchased. A total of 1.5 g TCD and 125 mg PVP were dissolved in 50 mL deionized water. A total of 2.6 g AgNO_3_ was dissolved in 40 mL deionized water. The AgNO_3_ solution was mixed with the TCD/PVP solution under magnetic stirring at a speed of 550 r/min for 1 h. Silver ink was prepared.

### 2.2. Fabrication of the Conductive Pattern by LDW

A 25-µm thick polyethylene terephthalate (PET) film was chosen as the flexible substrate of the flexible MAEs. The PET film was cleaned and treated by a low temperature oxygen plasma. Silver ink was uniformly coated on the PET film and dried for 1 h. A laser device (CNC4030, Wuhan Tuoli Automation Co., Ltd., Wuhan, China) was used to fabricate a conductive pattern on the PET film. The laser power, laser wavelength, and radius of the focused laser beam were 125 mW, 405 nm, and 50 µm, respectively. The conductive pattern was designed by AutoCAD (Autodesk, San Rafael, CA, USA). The PET film was fixed on the laser device with the silver ink side face down. The focused laser beam was focused on the interface of ink and PET film through the transparent PET film, and scanned along the designed path of conductive pattern under the control of the CNCC software of the laser device. The conductive pattern was sintered on the PET film. The left silver ink was washed away in deionized water. The conductive pattern on the PET film was dried under nitrogen.

### 2.3. Fabrication of the Conductive Pattern by LDW

The MRDL method was used for the one-step fabrication of the microneedle array from curable magneto-rheological fluid (CMRF) on the conductive pattern. The microneedle array was drawn in one step and formed from the CMRF droplets using a pillar array under the external magnetic field. The MRDL process has been well described in our previous paper [38].

### 2.4. Sputter Coating

A tattoo adhesive sheet (HPS LLC, El Paso, TX, USA) was taped to cover the conductive pattern as a mask, as shown in Figure 1c–d. A 10 nm Ti film and 100 nm Au film were coated on the surface of the microneedle array in sequence by a magnetron sputtering machine (MSP-3300, Jinshengweina Technology Co., Ltd., Beijing, China). Flexible MAE was finally fabricated. FDE without a microneedle array on the flexible substrate was also fabricated following the same process as flexible MAE. Flexible MAE and FDE can attach to the skin in a patch for bio-signal monitoring after peeling away the protective layer of the tattoo sheet. The adhesive layer is left on the flexible substrate, which can ensure good contact between the substrate and skin.

## 3. Bio-Signal Monitoring Tests

The bio-signal monitoring performance of the flexible MAE was evaluated in comparison with commercial wet electrodes (Ag/AgCl electrode, JK-1, Junkang Medical Supplies Ltd., Co., Shanghai, China) and FDE. Three healthy volunteers from 23 to 28 years old participated in the tests. The tests were repeated at least five times. This study was approved by the ethics committee of the Work Injury Rehabilitation Center of Guangdong Province (Approval No. AF/SC-07/2016.29). All volunteers provided written informed consent.

### 3.1. EII Test

**Figure 2:**
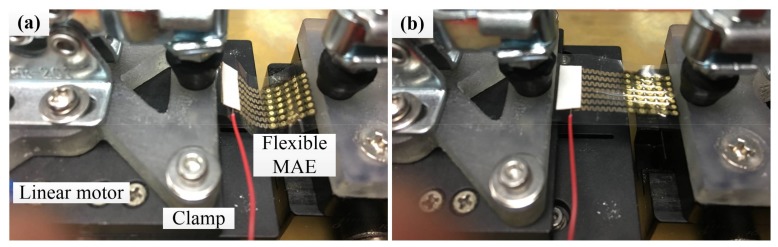
Setup for the flexible MAE bending test. (**a**) Flexible MAE in bending state, (**b**) Flexible MAE in stretching state.

A two-electrode measurement method was employed to record the EII [39]. The electrodes were placed on the left inner forearm and connected to a precision impedance analyzer (Agilent E4980A LCR Meter, Palo Alto, CA, USA). EIIs with a frequency from 20 Hz to 10 kHz was recorded. A setup for a flexible MAE bending test was developed, as shown in Figure 2. One end of the flexible MAE was fixed and the other was stretched with a linear motor (E-861, PI, Karlsruhe, Germany). The linear motor moved forward and back to bend the flexible MAE. The loading distance and speed was set as 10 mm and 1 mm/s, respectively. The flexible MAE was repeatedly bent for 50 cycles. The EII was measured before and after the bending test.

### 3.2. ECG Test

ECG signals in a static and dynamic state were recorded using the standard I-lead method [38]. Two working electrodes were patched on the left and right wrists, and the ground electrode was on the right ankle. The electrodes were connected to the ECG100C module of a Multipurpose Polygraph (MP150, BIOPAC, Goleta, CA, USA). The volunteers were asked to lie in bed in a static state and to mark time in the dynamic state.

### 3.3. EMG Test

Surface EMG signals of thumb motions were recorded. Two working electrodes were placed on the palm of the right hand with an interval of approximately 2 cm. The ground electrode was placed on the elbow. The electrodes were connected to the EMG100C module of the Multipurpose Polygraph. The volunteers were asked to perform three thumb motions, including move down, left and up, during the recording of the surface EMG. Each motion lasted 3 s.

### 3.4. EEG Test

A unipolar connection method was employed for the EEG test [33]. One working electrode was placed on the standard position (Fp1) of the 10–20 system, and both the other working electrode and ground electrode were placed on the left earlobe. The electrodes were connected to the EEG100C module of the Multipurpose Polygraph. Under the tester’s guide, the volunteers were asked to blink their eyes each second for 1 min during the eye blinking test, and to close and open alternate eyes with an interval of 2 s transition for 1 min during the closed and open eyes transition test.

## 4. Results and Discussion

### 4.1. Fabrication and Characterization of Flexibe MAE

**Figure 3:**
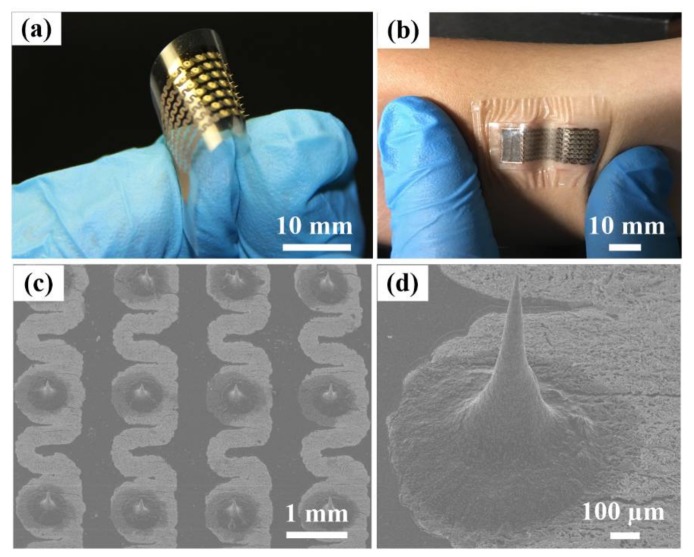
(**a**) Image of the flexible MAE, (**b**) image of the flexible MAE on the curved skin, (**c**) SEM (scanning electron microscope) image of the flexible MAE, and (**d**) SEM image of a microneedle.

A flexible MAE was fabricated, as shown in Figure 3a. The flexible MAE is flexible in that it can be easily bent in a circle. The flexible MAE can fully conform to the curved surface of human skin, even when subjected to a large skin deformation via the adhesive layer of tattoo sheet, as shown in Figure 3b. This close contact interface can effectively eliminate the relative shift between the flexible MAE and the skin, ensuring the reliable monitoring of bio-signals. The conductive pattern of the flexible MAE was designed and fabricated in a serpentine shape, as shown in Figure 3c. The serpentine design can enhance the mechanical deformability and prevent mechanical fracture by decreasing the induced strain [39]. This conductive pattern on the flexible substrate was directly written by focused laser beam. The focused laser beam passed through the transparent PET film and irradiated on the interface between the PET film and the silver ink layer. The silver ink was nonconductive. The silver nanoparticles were reduced from the silver ink after irradiation [19]. The silver nanoparticles were further sintered together, forming a conductive pattern on the flexible PET film along the scanning path of the laser beam. The conductive pattern was firmly bonded to the substrate due to the laser ablation at the interface between the PET substrate and pattern. Therefore, the silver conductive pattern could be simultaneously synthesized and written on the PET flexible substrate. The edges of the conductive pattern were relatively clear. The resistance of the serpentine pattern was less than 10 Ω. Above all, the conductive pattern can be custom designed and quickly fabricated by LDW.

A SEM image of the 5 × 7 microneedle array fabricated by the MRDL technique is shown in Figure 3c. Microneedles are arranged in an orderly manner on the circle pads. These microneedles were drawn in one step by the CMRF droplets on the flexible substrate by pillar array under an external magnetic field [38]. A pillar array hung with the dipped CMRF droplets was compressed onto the substrate. Subsequently, the pillar array was driven back and the compressed droplets were continuously stretched and became thinner, ultimately resulting in a break-up. A liquid microneedle array was formed and maintained on the substrate under the combination of gravity, surface tension and magnetic force, and was subsequently solidified. Ti/Au films were coated on the microneedle array surface for conductivity. The Ti film can enhance the adhesive strength of the Au film on the surface of the microneedle array [34]. Ti/Au films prevent the microneedle array from direct interaction with skin tissue, guaranteeing the biocompatibility of flexible MAE [40]. The SEM image of a microneedle is presented in Figure 3d. The shape of the microneedle is like a sharp cone. The microneedle surface is rough due to the iron particles dispersed in it, which may increase the contact area and friction between the microneedle and skin, forming a more stable interface. The average height, tip radius and base diameter of microneedle are about 500 ± 10 µm, 10 ± 5 µm, and 350 ± 15 µm, respectively. The height is appropriate for skin penetration [41,42]. The microneedle tip is extremely sharp, facilitating the skin penetration [43]. The diameter of the microneedle base is relatively large, enhancing the bonding strength of microneedle on the flexible substrate. It was demonstrated that the microneedle array fabricated by MRDL from CMRF showed good penetration performance in our previous study [37,38].

### 4.2. Bio-Signal Recording Performance

#### 4.2.1. EII Performance

**Figure 4:**
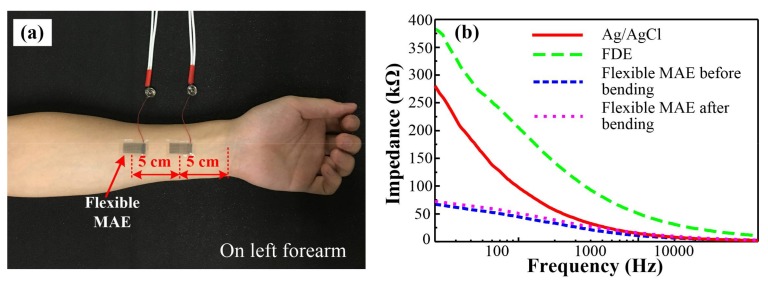
(**a**) EII measurement using flexible MAE, and (**b**) EII measured by the Ag/AgCl electrode, FDE, and flexible MAE before and after the bending test.

The EII measured by a flexible MAE on the forearm is shown in Figure 4a. The distance between two flexible MAEs is 5 cm. The EII measured by Ag/AgCl electrode, FDE, and flexible MAEs before and after bending tests is shown in Figure 4b. The EII decreased with the input current frequency from 20 Hz to 10 kHz. The measured EII consists of the resistance and capacitive resistance. The capacitive resistance decreased with the frequency. The EII measured by the flexible MAE was minimum, followed by the Ag/AgCl electrode, with the maximum measured by FDE. The possible reason for the results of the flexible MAE is that the penetration of microneedles into the skin tissue effectively eliminates the high impendence of the stratum corneum layer [7,22]. The EII measured by the flexible MAE increased slightly after the bending test, but was still lower than that of the Ag/AgCl electrode and the FDE. A local stress concentration may occur during the bending test, resulting in the formation of some small cracks in the conductive pattern and increasing the resistance of the flexible MAE. Above all, the EII measured by flexible MAE was the lowest even after 50 cycles of bending in comparison with the Ag/AgCl electrode and FDE.

#### 4.2.2. ECG Performance

**Figure 5:**
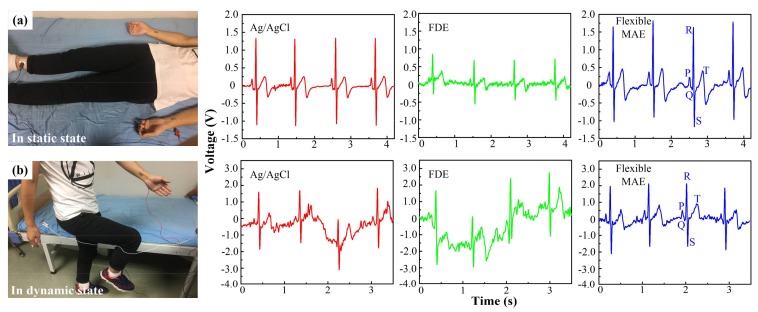
ECG signals recorded by the Ag/AgCl electrode, FDE and flexible MAE: (**a**) in the static state, and (**b**) in the dynamic state.

Static ECG signals in the time domain were recorded by the Ag/AgCl electrode, FDE and flexible MAE, as shown in Figure 5a. The characteristic ECG peaks (P, Q, R, S, and T) recorded by the flexible MAE and Ag/AgCl electrode are clearly visible, while obvious fluctuations and more noise can be observed in the ECG signal recorded by FDE. The EII measured by FDE was the highest and the contact interface between the FDE and skin was not stable. The ECG signal measured by the flexible MAE shows the highest magnitude. The dynamic ECG signals recorded by the Ag/AgCl electrode, FDE and flexible MAE are shown in Figure 5b. The volunteers were asked to mark time during the ECG recording. The ECG signals were seriously influenced by the motion due to the skin shift [7,43]. The flexible MAE exhibited the best dynamic ECG recording performance. Obvious signal drifts can be observed in the ECG signals recorded by the Ag/AgCl electrode and FDE. The QRS complex, P and T waves of the ECG signal recorded by the flexible MAE were more distinguishable with less noise. A possible reason for this is that the flexible MAE can pierce the skin and maintain a more stable contact interface between the electrodes and skin, weakening the effect of motion. It is more convenient to use the flexible MAE since no skin preparation is performed and no gel is used [44]. Therefore, flexible MAE is an alternative choice for ECG signal recording.

#### 4.2.3. EMG Performance

**Figure 6:**
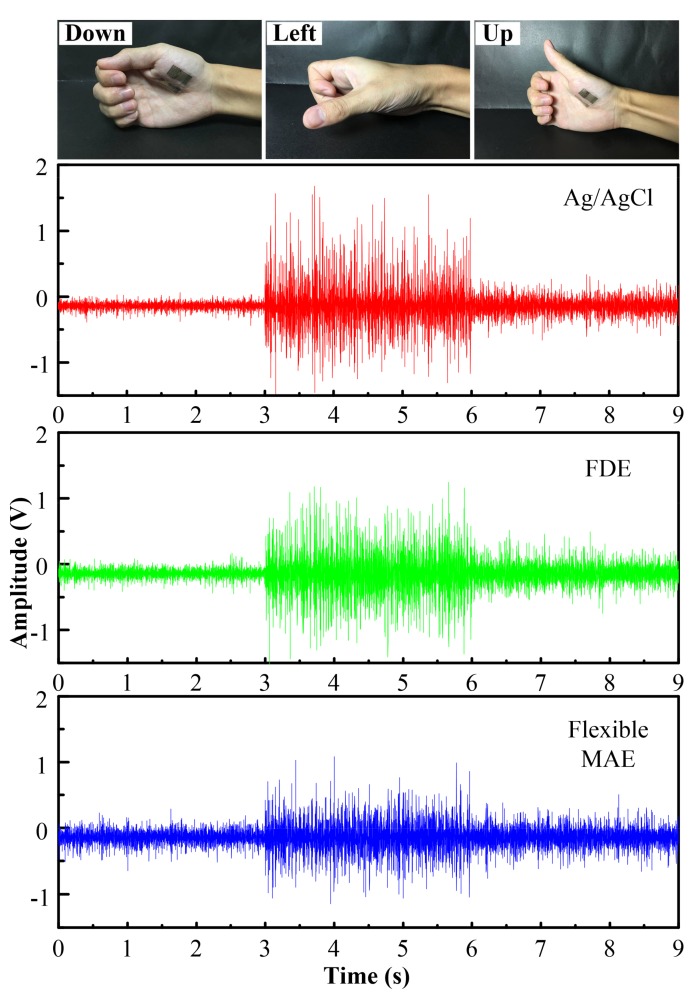
EMG signals of three thumb motions recorded by Ag/AgCl electrode, FDE and flexible MAE.

The muscle group of the thumb located in the palm is complex [31]. EMG signals of the thumb motions were recorded by the Ag/AgCl electrode, FDE and flexible MAE, as shown in Figure 6. The volunteers were asked to perform three thumb motions: move down, left, and up. The EMG signals fluctuate with the thumb motions. The signals required by the three electrodes show similar shapes and amplitudes for the three thumb motions. It demonstrates that the flexible MAE can trace the EMG signals along with the thumb motions with good fidelity. The contact area of the Ag/AgCl electrode (about 176 mm^2^) is 1.76 times larger than that of the flexible MAE (about 100 mm^2^). Thus, the flexible MAE is more suitable for application on small muscles. Furthermore, the microneedle distribution and number can be easily customized based on the distribution of the monitored muscles. The flexible MAE is flexible in that it can closely patch on the monitored muscles, achieving the selectivity of EMG signals and the elimination of crosstalk. This is a critical phenomenon in the case of several muscles present in small spaces or when the volunteers have small anthropometric dimensions [33]. Therefore, flexible MAE is a good selection for EMG recording.

#### 4.2.4. EEG Performance

**Figure 7:**
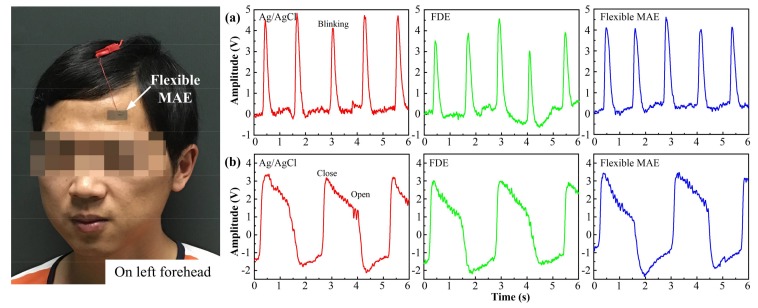
EEG signals recorded by the Ag/AgCl electrode, FDE and flexible MAE under: (**a**) the eyes blinking, and (**b**) the open and closed eyes transition.

The EEG signals of eye blinking recorded by the Ag/AgCl electrode, FDE and flexible MAE are shown in Figure 7a. The EEG signal measured by the flexible MAE is comparable in shape and amplitude to those of the Ag/AgCl electrode and FDE. The pulsed peaks can be obviously observed as the eyes blink. The frequency of eye blinking is about 1 Hz. Baseline drift can be found in the EEG signal recorded by FDE due to the skin shifting during eye blinking. The EEG signals of the eyes closing and opening transition were also recorded, as shown in Figure 7b. The EEG signals measured by the flexible MAE and the Ag/AgCl electrode were almost identical. The amplitude of the EEG signals varied along with the open and closed eyes being distinguishable. Flexible MAE can be directly used without skin preparation and the usage of gel, reducing the preparation time and avoiding skin allergies and the bother of gel drying. Therefore, flexible MAE is also suitable for EEG signal recording.

## 5. Conclusions

We introduced a simple process for fabricating a flexible MAE for wearable bio-signal recording. A serpentine conductive pattern was simultaneously synthesized and written on the PET flexible substrate by LDW. A liquid microneedle array on the conductive pattern was directly drawn from CMRF droplets by MRDL and solidified. The flexible MAE was fabricated as the microneedle surface was sputter coated with Ti/Au films. The flexible MAE conforms closely to the curved surface of human skin even when subjected to a large skin deformation. The microneedles had a sharp cone shape. The average height, tip radius, and base diameter of the microneedles were about 500 ± 10 µm, 10 ± 5 µm, and 350 ± 15 µm, respectively. The microneedle tip was sharp while the base was large, which facilitated skin penetration and enhanced the bonding strength of the microneedle on the substrate. The EII measured by the flexible MAE was the lowest even after 50 cycles of bending in comparison with the Ag/AgCl electrode and FDE. The flexible MAE was well able to record the ECG, EMG and EEG signals. The dynamic ECG signal recorded by the flexible MAE was the most distinguishable and introduced the least signal noise. Therefore, the flexible MAE is a promising electrode for bio-signal recording.
